# Draft Genome Sequence of Bacillus paralicheniformis F47, Isolated from an Algerian Salty Lake

**DOI:** 10.1128/genomeA.00190-18

**Published:** 2018-03-29

**Authors:** Mohamed Seghir Daas, Albert Remus R. Rosana, Jeella Z. Acedo, Malika Douzane, Farida Nateche, Salima Kebbouche-Gana, John C. Vederas

**Affiliations:** a Valcore Laboratory, Department of Biology, University M’Hamed Bougara of Boumerdes, Boumerdes, Algeria; b Food Technology Research Division, Institut National de la Recherche Agronomique d'Algérie, El Harrach, Algiers, Algeria; c Department of Chemistry, University of Alberta, Edmonton, Alberta, Canada; d Microbiology Group, Laboratory of Cellular and Molecular Biology, Faculty of Biological Sciences, University of Science and Technology–Houari Boumediene, Bab Ezzouar, Algiers, Algeria

## Abstract

Bacillus paralicheniformis F47 was isolated from a salty lake in Ain Baida-Ouargla, southern Algeria. The genome contains genes for the production of several bioactive secondary metabolites, including the siderophore bacillibactin, the lipopeptides fengycin, surfactin, and lichenysin, the antibiotics bacitracin and kanosamine, and a putative circular bacteriocin.

## GENOME ANNOUNCEMENT

Rhizophere-associated bacteria such as Bacillus spp. are able to suppress a variety of plant pathogens by producing antimicrobial secondary metabolites. For instance, Bacillus amyloliquefaciens subsp. amyloliquefaciens F11 can inhibit several Gram-positive and Gram-negative bacteria and fungal pathogens due to the production of antimicrobial compounds, including fengycin lipopeptides (1). Members of the B. cereus *sensu lato* group also produce a variety of bioactive small molecules such as lantibiotics and lipopeptides (2). Here, we report the draft genome of Bacillus paralicheniformis F47, isolated from a salty lake in southern Algeria, which contains multiple lipopeptides, siderophores, and a putative bacteriocin.

Genomic DNA was extracted using a DNeasy blood and tissue kit (Qiagen), as described previously (2). Sequencing libraries were created using the Nextera XT DNA library preparation kit, and sequencing was performed using the MiSeq reagent kit v2 (Illumina). *De novo* assembly of the 4,028,320-bp paired-end sequences (3.59 million reads) was generated using CLC Genomics Workbench v7.5.2. (CLC bio, Aarhus, Denmark), which resulted in 119 scaffolds, providing a coverage of 167× and G+C content of 46.2%. Gene prediction was performed using three automated annotation pipelines, Rapid Annotations using Subsystems Technology (RAST) v2.0 (3), the NCBI Prokaryotic Genome Annotation Pipeline (PGAP) (4), and the Joint Genome Institute-Integrated Microbial Genomes and Microbiomes (JGI-IMG/M) pipeline (5). Species identity was determined by calculating the average nucleotide identity (ANI) and alignment fraction (AF) using the Microbial Species Identifier (MiSI) calculator employed in the IMG/M system (6) against previously sequenced genomes in the IMG/M database. Strain identity was established by calculating the digital DNA:DNA hybridization (dDDH) using the Genome-to-Genome Distance Calculator (GGDC) 2.1 server (7). Secondary metabolites were predicted using antiSMASH v4 (8), and bacteriocins were predicted using BAGEL4 (http://bagel4.molgenrug.nl) (9). Genomic islands were predicted by using IslandViewer 4.0 (10), prophages by using PHASTER (11), and clustered regularly interspaced short palindromic repeats (CRISPRs) by using CRISPRfinder (12).

Genome annotation by the NCBI PGAP predicted 4,117 genes, including 4,026 coding sequences (CDSs), while RNA genes were composed of 11 rRNAs and 75 tRNAs. The genome of B. paralicheniformis F47 exhibits 99.26% ANI, 0.94 AF, and 70.54% dDDH with the type strain B. paralicheniformis KJ-16^T^ (13), supporting the placement of F47 in the species B. paralicheniformis. Conversely, the closest non-B. paralicheniformis species, Bacillus licheniformis ATCC 14580^T^, exhibits 94.69% ANI, 0.88 AF, and 10.96% dDDH. Two putative CRISPR modules, one prophage, and three genomic islands were discovered in the genome.

The F47 genome contains several gene clusters with homology to known lipopeptide biosynthetic operons, including fengycin (*fenABCDE*) (1), surfactin (*srfADCB*) (14), and lichenysin (*lchAA* and *lchAB*) (15). The operons for the antibiotics bacitracin (scaffolds 11, 43, and 103) and kanosamine (*kabABC*) (16) were detected in the genome. Two siderophore gene clusters were also predicted in the genome, including bacillibactin and a putative anthrachelin (17). Lastly, a gene cluster of a putative circular bacteriocin was identified.

### Accession number(s).

This whole-genome shotgun project has been deposited in DDBJ/ENA/GenBank and JGI-IMG/M under the accession numbers MYFI00000000 and Ga0181491, respectively.
